# Differential Immune Response against Recombinant *Leishmania donovani* Peroxidoxin 1 and Peroxidoxin 2 Proteins in BALB/c Mice

**DOI:** 10.1155/2015/348401

**Published:** 2015-08-25

**Authors:** Nada S. Daifalla, Abebe Genetu Bayih, Lashitew Gedamu

**Affiliations:** ^1^Department of Biological Sciences, University of Calgary, Room 374, 2500 University Drive NW, Calgary, AB, Canada T2N 1N4; ^2^The Forsyth Institute, Cambridge, MA 02142, USA

## Abstract

We assessed the immune response against recombinant proteins of two related, albeit functionally different, peroxidoxins from *Leishmania donovani*: peroxidoxin 1 (LdPxn1) and peroxidoxin 2 (LdPxn2) in BALB/c mice. We also evaluated the effect of coadministration of TLR agonists (CpG ODN and GLA-SE) on the antigen-specific immune response. Immunization with recombinant LdPxn1 alone induced a predominantly Th2 type immune response that is associated with the production of high level of IgG1 and no IgG2a isotype while rLdPxn2 resulted in a mixed Th1/Th2 response characterized by the production of antigen-specific IgG2a in addition to IgG1 isotype. Antigen-stimulated spleen cells from mice that were immunized with rLdPxn1 produced low level of IL-10 and IL-4 and no IFN-*γ*, whereas cells from mice immunized with rLdPxn2 secreted high level of IFN-*γ*, low IL-4, and no IL-10. Coadministration of CpG ODN or GLA-SE with rLdPxn1 skewed the immune response towards a Th 1 type as indicated by robust production of IgG2a isotype. Furthermore, the presence of TLR agonists together with rLdPxn1 antigen enhanced the production of IFN-*γ* and to a lesser extent of IL-10. TLR agonists also enhanced a more polarized Th 1 type immune response against rLdPxn2.

## 1. Introduction

Infection by parasites of the genus* Leishmania* results in a chronic disease known as leishmaniasis. It is transmitted when an infected female phlebotomine sandfly injects the metacyclic promastigotes into the host during a blood meal. The flagellated promastigotes are taken by macrophages where they transform into aflagellated amastigotes that multiply and disseminate the infection [1]. The outcome of* Leishmania* infection depends on the species of* Leishmania* as well as the host immune response. Clinical manifestation of leishmaniasis ranges from self-healing cutaneous form to fatal visceral disease [2]. The disease is prevalent worldwide infecting millions of people in more than 90 countries in the tropics, subtropics, and southern Europe (Center for Disease Control and Prevention, http://www.cdc.gov/parasites/leishmaniasis/). About 1.3 million new cases and about 30000 deaths are recorded each year with the majority of these cases occurring in poor regions of the world (World Health Organization, http://www.who.int/mediacentre/factsheets/fs375/en/, [3]) where the afflicted populations have low accessibility to health care. Chemotherapy is available but its usefulness is compromised by toxicity of some drugs and drug resistance by the parasite [4]. In addition, the emergence of* Leishmania*/HIV coinfection compounded the problem. Concomitant infection with HIV increases the cases of active VL in otherwise asymptomatic individuals by 100 to 1000 times and it increases the likelihood of drug toxicity as well as relapse of the disease [5].

Experimental studies have shown that protection against leishmaniasis is mediated by T helper 1 (Th1) type CD4^+^ cells that produce a high level of interferon gamma (IFN-*γ*) and tumor necrosis factor alpha (TNF-*α*) whereas progression of the disease is associated with Th2 type CD4^+^ cells which produce IL-4, IL-5, IL-10, and IL-13 [6–9]. The Th1 and Th2 cells have differential capabilities in stimulating B cells to secrete different antibody isotypes where Th1 type cells elicit IgG2a antibody production and Th2 type cells induce IgG1 antibody secretion [10]. This differential effect is brought about by the regulatory effect of cytokines on the immunoglobulin isotype switching.* In vitro* studies have shown that IL-4 and IFN-*γ* stimulate the production of IgG1 and IgG2a, respectively [11, 12].

*Leishmania* parasites are highly successful in parasitizing macrophage cells which are otherwise hostile to pathogens. Generally, uptake of pathogenic organisms by macrophages results in oxidative burst which is associated with the production of reactive oxygen species (ROS) such as superoxide radical (O_2_^−•^), hydrogen peroxide (H_2_O_2_), and hydroxyl anion (OH^−^) and reactive nitrogen species (RNS) including nitric oxide (NO). These reactive species are highly destructive to the infecting pathogen and they can interact with each other forming more potent oxidants such as peroxynitrite (ONOO^−^) [13].

**Figure 1:**
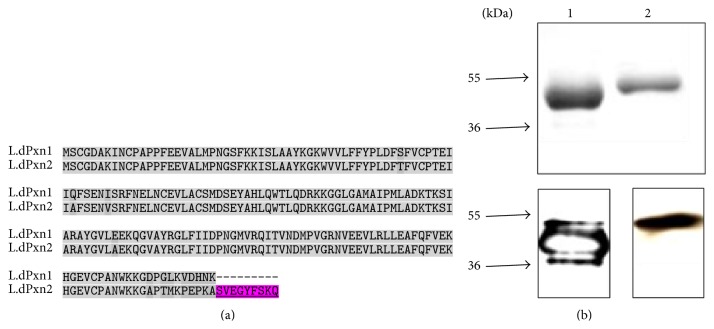
(a) Sequence comparison of* Leishmania donovani* Pxn1 and Pxn2. Alignment of amino acid sequence depicts the high homology between LdPxn1 and LdPxn2. Highlighted areas show positions of mismatch. LdPxn2 possesses extra 9 amino acids at the carboxy terminus (underlined) that are missing from LdPxn1. (b) SDS-PAGE and western blot of rLdPxn1 and rLdPxn2 proteins. One microgram per lane of rLdPxn1 (lane 1) and rLdPxn2 (lane 2) was separated on a 12% SDS-PAGE and stained with Coomassie blue, top. The separated samples were transferred to Hybond-P membrane and were probed with pooled sera from mice immunized with the respective recombinant protein, bottom. Molecular weight in kDa is shown on the left.

One of the evasive mechanisms used by* Leishmania* parasites to bypass the microbicidal effect of free radicals produced by macrophages is the expression of antioxidant enzymes known as peroxidoxins. These enzymes are conserved and highly abundant proteins in almost all living organisms which suggest essential function in oxidative homeostasis. It has been shown that peroxidoxins from different organisms including* Leishmania* are important in the protection of these organisms against oxidative stress [14–16]. We isolated and characterized three peroxidoxins as part of a multigene family from* L. donovani* complex: Pxn1, Pxn2, and Pxn3 [14, 17]. Both Pxn1 and Pxn2 are cytosolic whereas Pxn3 is predicted to be glycosomal. A fourth mitochondrial peroxidoxin, Pxn4, has also been identified in* L. donovani* [18]. In addition to the common localization of Pxn1 and Pxn2 in the cytoplasm, the two proteins have 89.4% homology. The difference between these two proteins is brought about by an extra 9 amino acids at the carboxy terminus of Pxn2 plus few nucleotide mismatches along the entire sequence [14, 17] (Figure 1(a)). Despite the high similarity between LdPxn1 and LdPxn2 at the amino acid level, there are striking differences between the proteins encoded by the two genes. Unlike LdPxn1, which is upregulated during the amastigote stage, LdPxn2 is expressed at high levels during the promastigote stage and the expression declines towards the amastigote stage. In addition, while recombinant LdPxn1 protein has been shown to detoxify various free radicals including ROS and RNS, LdPxn2 can only detoxify H_2_O_2_ [14].

In this study, we assessed the immune responses against LdPxn1 and LdPxn2 as recombinant GST-fusion proteins in BALB/c mice to test if the differences observed in gene expression and functionality between these two antigens are paralleled by different immune response profile. In addition, we evaluated the immune response against these proteins in the presence of two Th1 adjuvants: bacterial CpG oligodeoxynucleotide (CpG ODN) and glucopyranosyl lipid A in a stable emulsion (GLA-SE), which are Toll-like receptor 9 (TLR-9) and TLR-4 agonists, respectively. Our results indicate that mice immunization with LdPxn1 induces a predominant Th2 type response, whereas immunization with LdPxn2 stimulates a mixed Th1/Th2 response. Our data also show that repeated injections with coadministration of Th1-adjuvants enhanced the immune response against LdPxn1 and LdPxn2 which is more biased towards Th1 type.

## 2. Materials and Methods

### 2.1. Mice

Female BALB/c mice (4–6 weeks old) were purchased from Charles River Laboratories (QC, Canada) and were housed in a specific pathogen-free facility at the University of Calgary and provided water and food* ad libitum*. Mice were acclimatized for one week and randomly distributed into experimental groups and controls. Animal protocols were approved by the Life and Environmental Sciences Animal Care Committee (LESACC) of the University of Calgary, Alberta, Canada.

### 2.2. Cloning, Expression, and Purification of Recombinant LdPxn1 and LdPxn2 in* E. coli*

Cloning of LdPxn1 and LdPxn2 as GST-fusion proteins was performed by using the prokaryotic expression vector, pGEX-2T (Amersham Pharmacia Biotech) following the procedure described previously [17]. Briefly, the coding regions of LdPxn1 and LdPxn2 were amplified by PCR using specific primers. The amplified fragments were then cloned into pGEX-2T vector. To express the recombinant proteins, transformed* E. coli* BL21 (DE3) cells were grown in a 37°C shaker overnight in Luria-Bertani (LB) broth in the presence of 100 *μ*g/mL ampicillin. The cultures were induced with 0.2 mM isopropyl beta-D-thiogalactoside (IPTG) and continued to grow for 3–6 hours. Fusion proteins, GST-LdPxn1 and GST-LdPxn2, were harvested by sonication and passing over a glutathione-agarose resin column (Sigma) as described by Smith and Johnson [19]. Endotoxins were removed using Detoxi-Gel Affinity Pak prepacked columns following the manufacture's instruction (Pierce Biotechnology, USA). Endotoxin level of protein samples was measured at the Infectious Disease Research Institute (Seattle, USA) using Limulus Amebocyte Lysate (LAL) assay. Samples of endotoxin levels <10 EU/mg protein were used.

### 2.3. Immunization

**Figure 2:**
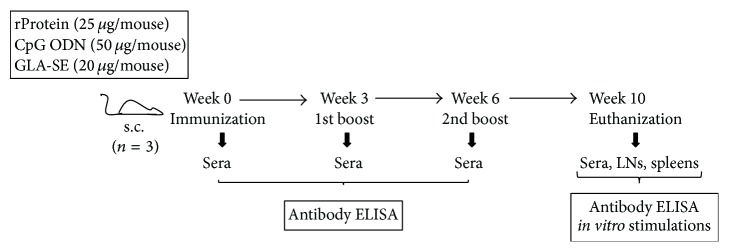
Schematic representation of the immunization protocol. Six- to 8-week-old female BALB/c mice were immunized s.c. in the hind foot pad with recombinant LdPxn1 or LdPxn2 protein (25 ug/mouse) with or without CpG ODN (50 ug/mouse) or GLA-SE (20 ug/mouse) adjuvants. Mice were boosted twice in 3 weeks interval. Sera were collected at each time of injection. Four weeks after the last boost mice were euthanized and sera, lymph nodes, and spleens were collected. Samples were used for antibody and cytokine analysis using standard protocols.

Immunization protocol is schematically represented in Figure 2. Mice were randomly divided into groups of three and were immunized subcutaneously (s.c.) with recombinant LdPxn1, rLdPxn1 plus CpG ODN, rLdPxn1 plus GLA-SE, rLdPxn2, rLdPxn2 plus CpG ODN, and rLdPxn2 plus GLA-SE. Recombinant proteins, CpG ODN 1826 (Coley Pharmaceutical Group, Canada) and GLA-SE (Infectious Disease Research Institute, Seattle, USA), were given at 25, 50, and 20 *μ*g/mouse, respectively. Two booster injections were given in three-week interval. Sera were isolated from blood (collected every three weeks starting from the time of first immunization (week 0) until the time of euthanization) and stored at –20°C. Mice were euthanized four weeks after the last boost, and lymph node and spleens were aseptically harvested and processed for the isolation of single cell suspensions. The isolated lymph node and spleen cells were used for* in vitro* antigen stimulation experiments.

### 2.4. Western Blotting

For western blotting, 1 *μ*g of each of the recombinant proteins was separated by SDS-PAGE and transferred to Hybond-P membrane (GE Healthcare, QC, Canada). The membrane was blocked with 5% skim milk dissolved in phosphate buffered saline (PBS) containing 0.05% tween-20 (PBS-T) for 2 hr at room temperature. Then, it was incubated overnight at 4°C with mice serum that was collected four weeks after the last immunization with the respective antigen. After washing three times with PBS-T, the membrane was incubated with a horseradish peroxidase-conjugated anti-mouse IgG (GE Healthcare, QC, Canada) for 45 min at room temperature (RT) followed by three washing steps. Immunoreactivity was detected by chemiluminescence using ECL reagents following the manufacturer's instructions (GE Healthcare, QC, Canada).

### 2.5. Antibody Measurement

The presence of antibody specific to LdPxn1 and LdPxn2 in serum samples was determined by enzyme-linked immunosorbent assay (ELISA). Briefly, 96-well microtiter plates (Sarstedt, USA) were coated overnight at 4°C with 1 *μ*g/mL recombinant protein in bicarbonate buffer, pH 9.6. The plates were blocked with 5% (w/v) skim milk in PBS-T for 1 hr at RT. After three washes with PBS-T, 100 *μ*l/well of sera diluted 1 : 100 in blocking buffer was added to the plates and incubated for 1 hr at RT. After washing, 100 *μ*l/well of biotinylated goat anti-mouse IgG1 or IgG2a antibody was added to the wells and incubated for 1 hr at RT followed by 1 hr incubation with streptavidin-HRP. The reaction was then developed by adding 100 *μ*l/well TMB (3,3′,5,5′-tetramethylbenzidine) substrate (BD Biosciences, ON, Canada). After the reaction was stopped by adding 50 *μ*l/well 1 N H_2_SO_4_, the plates were read at 450 nm in a microplate reader (Molecular Devices, USA).

### 2.6. *In Vitro* Antigen Stimulation and Cytokine Measurement

Mice were euthanized 4 weeks after the last immunization and lymph node and spleen cells were isolated as described previously [20]. Cells from lymph nodes of mice from the same group were pooled before* in vitro* stimulation. For stimulation assays, cells from individual spleens or from pooled lymph nodes were dispensed at 2 × 10^5^ cell/100 *μ*L media/well in 96-well flat bottomed tissue culture plates (Sarstedt, USA) and incubated with 2 or 10 *μ*g/mL of recombinant protein in complete medium (RPMI-1640 supplemented with 10% FBS, 2 mM L-glutamine, 25 mM HEPES, penicillin (100 U/ml) plus streptomycin (100 lg/mL), and 50 *μ*M *β*-Mercaptoethanol) for 72 hr at 37°C in a humidified incubator with 5% CO_2_. Cells were also incubated with 5 *μ*g/ml concanavalin A (ConA) or with medium alone as positive and negative control, respectively. Culture supernatants were collected and cytokine production was measured using cytokine ELISA kits as per the manufacturer's instructions (BD Bioscience, ON, Canada) as described previously [20]. The amount of IFN-*γ* and IL-10 produced by lymph node or spleen cells was expressed as ng/ml. In addition, the production of IL-4 was measured in spleen cells and was expressed as pg/mL.

### 2.7. Statistics

Data are expressed as the mean ± standard error of the mean (S.E.M.). Statistical analysis was performed using Student's *t*-test. *P* value of less than 0.05 was considered statistically significant.

## 3. Results

### 3.1. Recognition of LdPxn1 and LdPxn2 by Immune Sera

To demonstrate the immunoreactivity of recombinant LdPxn1 and LdPxn2 in BALB/c mice, we tested the interaction between sera collected from immunized mice and the respective recombinant protein by western blot analysis. As depicted in Figure 1(b), mice immune sera bound to the respective recombinant protein immobilized onto the membranes as indicated by the prominent bands of the expected molecular size of the GST-fused proteins. This indicates that both proteins are immunogenic in BALB/c mice.

### 3.2. Comparative Analysis of Humoral Immune Response to Recombinant LdPxn1 and LdPxn2 Proteins

To analyze the isotype profile of antibody response in mice immunized with rLdPxn1 or rLdPxn2, we measured antigen specific IgG1 and IgG2a isotypes in sera collected at different time points after immunization. In addition, we calculated the ratio of IgG2a to IgG1 as a surrogate marker for Th1 type immune response.

**Figure 3:**
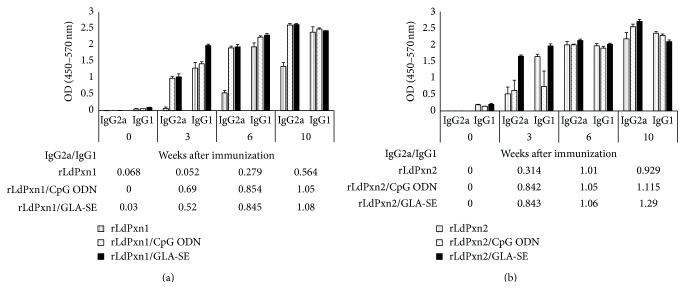
Anti-LdPxn1 and -LdPxn2 antibodies in immunized mice. Mice were immunized subcutaneously with recombinant LdPxn1 or LdPxn2 proteins with or without CpG ODN or GLA-SE. Mice were boosted twice in 3-week intervals. The levels of IgG1 and IgG2a isotypes were measured on sera collected at different time points using ELISA. Data are presented as the mean OD ± S.E.M. of IgG1 and IgG2a of sera from mice immunized with rLdPxn1 (a) and rLdPxn2 (b). The IgG2a/IgG1 ratios are shown in tables below each figure.

As shown in Figure 3(a), immunization of mice with rLdPxn1 by itself stimulated a high level of IgG1 isotype and barely detectable amount of IgG2a at 3 weeks after the first immunization. The amount of specific IgG2a stimulated in this group increased upon booster immunization; however it remains significantly lower than the amount of IgG1 (*P* < 0.05). Concomitant injection of CpG ODN or GLA-SE with rLdPxn1 triggered a high level of IgG1 and more importantly a high level of IgG2a as well (Figure 3(a)). Similar to immunization with rLdPxn1 alone, the production of anti-rLdPxn1 antibodies in mice immunized with rLdPxn1 plus adjuvants was augmented by booster immunization (Figure 3(a)). The augmentation effect of booster injections together with the presence of TLR agonists in the immunization protocol resulted in the induction of IgG2a level as high as IgG1 four weeks after the last boost.

In contrast to rLdPxn1, rLdPxn2 alone was able to induce specific IgG2a production, in addition to IgG1 isotype, as early as 3 weeks after the first immunization (Figure 3(b)). The level of both isotypes was enhanced by booster immunizations in this group and the level of IgG2a was comparable to the level of IgG1 after the second injection, that is, the first boost (Figure 3(b)). Coadministration of CpG ODN or GLA-SE adjuvants with rLdPxn2 resulted in the production of high and comparable levels of both IgG2a and IgG1 isotypes as early as 3 weeks after the first boost (week 6) (Figure 3(b)). Coadministration of CpG ODN with rLdpxn2 induced lower antibody response as compared to GLA-SE after the first injection. However, the level of both isotypes in all groups receiving rLdPxn2 was comparable after the second booster immunization. At this time point, the level of IgG2a was slightly less or slightly more than IgG1 in the group immunized with rLdPxn2 alone or rLdPxn2 plus adjuvant, respectively.

These results indicate that, in BALB/c mice, priming with recombinant LdPxn1 induces a predominantly Th2 response (IgG2a/IgG1 ratio of 0.052) whereas priming with recombinant LdPxn2 stimulates a mixed Th1/Th2 response (IgG2a/IgG1 ratio of 0.314). The data also reveal that, four weeks after the second boost, the ratio of IgG2a/IgG1 increased to 0.564 and 0.929 for rLdPxn1 and rLdPxn2, respectively. These results suggest that booster immunization can enhance the immune response against rLdPxn1 and rLdPxn2. Our findings also show that CpG ODN and GLA-SE adjuvants have the capacity to skew the immune response against rLdPxn1 and rLdPxn2 toward a more Th1 type (IgG2a/IgG1 ratio > 1.0 after the last boost).

### 3.3. Antigen Specific Cellular Immune Response

To understand the type of cell-mediated immune response (CMI) against rLdPxn1 and rLdPxn2, we measured the level of IFN-*γ* and IL-10 in antigen-stimulated lymph node cells and the level of IFN-*γ*, IL-10, and IL-4 in the spleen cells of immunized mice. Lymph node cells from mice in each group were pooled and stimulated* in vitro* with 10 *μ*g/mL of recombinant proteins whereas spleen cells from individual mouse were stimulated with 2 or 10 *μ*g/mL of recombinant proteins. No stimulation or stimulation with 5 *μ*g/mL Con A was added as negative and positive controls, respectively. Culture supernatants were collected 72 hr later and the amount of cytokines was determined by ELISA.

**Figure 4:**
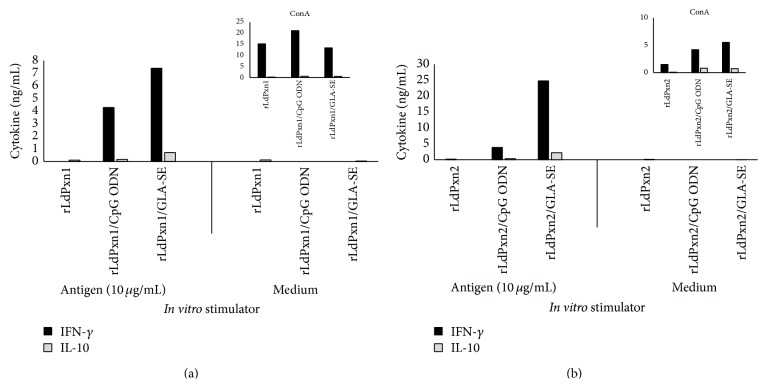
Cytokine responses in lymph node cells of rLdPxn1 and rLdPxn2 immunized mice. Mice were immunized subcutaneously three times at three-week intervals with rLdPxn1 or rLdPxn2 with or without CpG ODN or GLA-SE. Four weeks after the last immunization, cells from pooled lymph nodes were stimulated* in vitro* with the respective antigen (10 *μ*g/mL) or ConA (5 *μ*g/mL). The release of IFN-*γ* and IL-10 in mice immunized with rLdPxn1 or rLdPxn2 was measured in supernatants after 72 hr of* in vitro* stimulation at 37°C. Results are presented as the amount of IFN-*γ* (ng/mL) and IL-10 (ng/mL) for rLdPxn1 (a) and rLdPxn2 (b).

There was no spontaneous release of cytokines by unstimulated lymph node cells in any of the groups (Figures 4(a) and 4(b)). No detectable cytokine was released by lymph node cells from mice immunized with the recombinant proteins alone (Figures 4(a) and 4(b)). However, immunization of mice with the recombinant proteins in the presence of TLR agonists resulted in the production of a high level of IFN-*γ* and a low level of IL-10 (Figures 4(a) and 4(b)). While coadministration of CpG ODN triggered the production of similar amounts of IFN-*γ* in mice immunized with rLdPxn1 or rLdPxn2, GLA-SE stimulated the release of more IFN-*γ* in the group receiving rLdPxn2. The amount of IFN-*γ* produced by lymph node cells of mice from this group (Figure 4(b)) was more than 3-fold higher than the level produced by lymph node cells from mice immunized with rLdPxn1 alone (Figure 4(a)). Interestingly, CpG ODN and GLA-SE triggered the production of lower amount of IL-10 in mice receiving rLdPxn1 as compared to rLdPxn2 (Figures 4(a) and 4(b)). Lymph node cells stimulated with ConA mitogen produced comparable levels of cytokines (Figures 4(a) and 4(b) in sets) with the exception of the group immunized with rLdPxn2 alone which produces lower level of cytokines (Figure 4(b) in set). The results of cytokine analyses in lymph node cells demonstrate that rLdPxn1 and rLdPxn2 can stimulate lymph node cells of immunized mice to produce cytokines only in the presence of adjuvants. The results also show that CpG ODN and GLA-SE adjuvants favor a Th1 type response against the two antigens as indicated by the high IFN-*γ*/IL-10 ratios (Table 1). Moreover, the results show that while coadministration of GLA-SE exerts comparable effect on both antigens as indicated by comparable ratios of IFN-*γ*/IL-10 (Table 1), CpG ODN induces stronger Th1 in mice receiving rLdPxn1 as compared to rLdPxn2 (IFN-*γ*/IL-10 ratio of 24.74 and 10.83 for rLdPxn1 and rLdPxn2, resp.) (Table 1).

**Figure 5:**
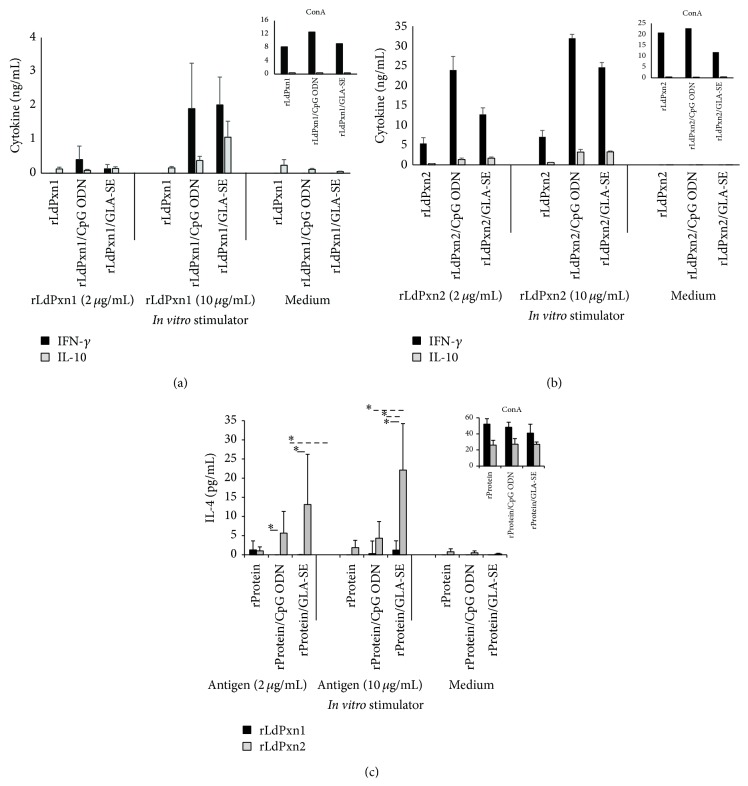
Cytokine responses in spleen cells of rLdPxn1 and rLdPxn2 immunized mice. Mice were immunized s.c. three times at three-week intervals with rLdPxn1 or rLdPxn2 with or without CpG ODN or GLA-SE. Four weeks after the last immunization, spleen cells were prepared and* in vitro* stimulated with the respective antigen (2 and 10 *μ*g/mL) or ConA (5 *μ*g/mL). The release of IFN-*γ* (ng/mL), IL-10 (ng/mL), and IL-4 (pg/mL) in immunized mice was measured in supernatants after 72 hr of* in vitro* stimulation at 37°C. Results are presented as the amount of IFN-*γ* (ng/mL) and IL-10 (ng/mL) for rLdPxn1 (a) and rLdPxn2 (b) or the amount of IL-4 (pg/mL) of rLdPxn1 and rLdPxn2 (c).

Production of IFN-*γ*, IL-10, and IL-4 by spleen cells of immunized mice is depicted in Figure 5.

Spleen cells from mice immunized with rLdPxn1 by itself did not produce any detectable level of IFN-*γ* and very low level of IL-10 upon i*n vitro* stimulation with 2 or 10 *μ*g/ml rLdPxn1 (Figure 5(a)). In contrast, immunization with rLdPxn2 alone was able to stimulate mice spleen cells to produce considerable amount of IFN-*γ* but low IL-10 in* in vitro* recall experiments with 2 and 10 ug/ml rLdPxn2 (Figure 5(b)). Both rLdPxn1 and rLdPxn2 stimulated the production of a low level of IL-4 in spleen cells of immunized mice when stimulated* in vitro* with 2 *μ*g/mL of the respective protein. However, IL-4 production was only detected in spleen cells from the group receiving rLdPxn2 upon stimulation with 10 *μ*g/mL (Figure 5(c)).

Administration of rLdPxn1 in the presence of CpG ODN results in the production of a low level of IFN-*γ* and almost no IL-10 when the spleen cells were stimulated with 2 *μ*g/ml of the antigen (Figure 5(a)). At this concentration, stimulated spleen cells from mice receiving rLdPxn1 plus GLA-SE produced low but comparable levels of IFN-*γ* and IL-10. The level of IFN-*γ* and IL-10 produced by spleen cells from mice that received rLdPxn1 plus the adjuvants was dose-dependent with the production of higher levels of each cytokine upon stimulation with 10 *µ*g/mL of the recombinant protein (Figure 5(a)).

*In vitro* stimulation of spleen cells from mice immunized with rLdPxn2 plus CpG ODN or GLA-SE with 2 and 10 *μ*g/ml produced a high level of IFN-*γ* and a low IL-10 (Figure 4(b)). Interestingly, as shown in Figure 5(c), the presence of the TLR agonists in the immunization protocol induced the production of a higher level of IL-4 by the spleen cells from mice immunized with rLdPxn2 as compared to low or none from those immunized with rLdPxn1. Spontaneous release of a low level of IL-10 by spleen cells from mice receiving rLdPxn1 by itself or with CpG ODN was observed (Figure 5(a)) as well as a low level of IL-4 by spleen cells from mice immunized with rLdPxn2 alone or plus CpG ODN (Figure 5(c)).

These results show that rLdPxn1 alone stimulates a weak cell-mediated immunity in the spleens of immunized mice as indicated by the low level or absence of detectable cytokines in* in vitro* recall experiments. Administration of rLdPxn1 in the presence of CpG ODN or GLA-SE increased the immune response with higher IFN-*γ*/IL-10 ratio (3.78 and 2.03 for rLdPxn1-CPG ODN and rLdPxn1 GLA-SE, resp.) (Table 2) and low IL-4 (Figure 5(c)). On the other hand, immunization of mice with rLdPxn2, in the presence or absence of adjuvants, results in a mixed Th1/Th2 type response in spleen cells of immunized mice associated with high IFN-*γ*/IL-10 ratio (12.82, 10.88, and 7.55 for rLdPxn2, rLdPxn2 CpG ODN, and rLdPxn2 GLA-SE, resp.) (Table 2) and high level of IL-4 (Figure 5(c)). This observation indicates that, independently of the adjuvant use, rLdPxn2 is capable of inducing a mixed Th1/Th2 response biased toward a Th1 type.

## 4. Discussion

In this study, we report differential immune responses against two cytosolic* Leishmania donovani* peroxidoxins: LdPxn1 and LdPxn2. These two antioxidants are highly homologous, yet they are differentially expressed. The expression of LdPxn1 is upregulated during the mammalian amastigote stage whereas LdPxn2 is highly abundant in the promastigote stage [17]. In addition, LdPxn1 and LdPxn2 are functionally different; LdPxn1 has been found to detoxify a wide range of reactive species (ROS and RNS) while LdPxn2 can only neutralize H_2_O_2_ [14]. The main focus of this work was to examine and compare the humoral and cellular immune responses against recombinant LdPxn1 and LdPxn2 GST-fusion proteins in BALB/c mice and to investigate the potential of two TLR agonists as adjuvants that can be used with these recombinant proteins.

Our findings show that recombinant LdPxn1 protein induces a predominant Th2 type immune response in mice, whereas rLdPxn2 stimulates a mixed Th1/Th2 response biased toward a Th1 type. Our data also demonstrate that coadministration of CpG ODN and GLA-SE favors the stimulation of a polarized Th1 type response with increased ratios of IgG2a/IgG1 and IFN-*γ*/IL-10. This finding is not unprecedented since several previous studies have also shown the ability of these TLR-based adjuvants to stimulate a protective Th1 response against* Leishmania* antigens [21–27].

The mechanism by which recombinant LdPxn1 and LdPxn2 stimulate different immune responses in BALB/c mice was not investigated in this study and it remains to be defined; however, possible explanations are discussed below.

One possibility is that rLdPxn1 and rLdPxn2 are recognized by different TLRs which may result in the stimulation of different effector mechanisms. It has been reported that a mycobacterial early secreted antigenic target protein 6 (ESAT-6) can directly bind to Toll-like receptor 2 and modulate the host immune response [28, 29]. Studies have suggested that lymphocyte-derived cytokines released following TLR ligation can regulate T helper cell differentiation and the type of induced immunity (reviewed in [30]). Additional explanation is that the two antigens may differ in their intracellular trafficking such that they undergo different processing and presentation with major histocompatibility complex (MHC) molecules by antigen presenting cells (APCs). This possibility has been proposed as a possible cause for the differences in immune responses triggered against* L. major* TSA and LmsTI1 antigens [31]. Dendritic cells (DCs) are professional antigen presenting cells capable of stimulating T-cell activation [32]. Studies have shown the participation of these cells in Th1/Th2 polarization through differential production of IL-12 and IL-10 [33], as well as IFN-*γ* [34]. Thus, interaction of antigens with DCs is central to the priming and differentiation of T cells.

Also requiring further study is the relationship between rLdPxn1 and rLdPxn2 structures and the immune response as these two antigens may have structural differences, in particular differences in their antigenic epitopes that might affect the humoral immune responses generated against them.* Leishmania* possesses the typical 2-Cys peroxidoxins which have two conserved cysteine (Cys) residues the peroxidatic cysteine Cys47 located at the N-terminus and the resolving cysteine Cys170 placed near the carboxyl terminus [35, 36]. In general, active peroxidoxins exist as homodimers arranged in a head-to-tail orientation such that the N-terminus cysteine of one monomer is juxtaposed with the C-terminus cysteine on the opposing subunit. The transition of peroxidoxins from the reduced to the oxidized state is commonly associated with a conformational change involving the C-terminus tail. Although the structure of LdPxn1 and LdPxn2 is not available, we anticipate that, following conformational changes (resulting from changes in redox state or from antigen processing), the LdPxn2 C-terminus amino acid extension, composed of the terminal 9 amino acids plus few up-stream amino acids which also exhibit differences from LdPxn1, might present different epitopes and consequently stimulates immune response distinct from LdPxn1. A simple experiment to examine the contribution of LdPxn2 C-terminus extension in shaping the immune response can be done by testing its immunoreactivity in mice. Alternatively, mice immune response to a mutated LdPxn2 molecule depleted of the C-terminus extension or of LdPxn1 molecule to which the LdPxn2 C-terminus extension is introduced can be tested and compared to the immune response against the original molecules.

It is important to note that LdPxn1 and LdPxn2 were examined in the form of recombinant GST-fusion proteins. It has been documented that the immune responses generated against GST-fusion proteins are greatly affected by the carrier portion of the protein as well as the adjuvant used [37, 38]. Moreover, it has been suggested that GST fusion may cause conformational changes of proteins permitted by the flexible linker region [39]. Despite the fact that the rLdPxn1 and rLdPxn2 used in this study were generated in the same way, we believe that it is important to analyze the possible effects that GST fusion may have on the immune response against these proteins.

Our future studies will focus on elucidating the possible mechanisms that regulate mice immune responses against LdPxn1 and LdPxn2. We believe that it is important to understand the potential mechanisms by which these antigens interact with the host immune system to shed light on the factors behind the difference in the immune response to seemingly similar antigens.

## 5. Conclusions

In conclusion, we observed distinct immune response against rLdPxn1 and rLdPxn2 in BALB/c mice. Recombinant LdPxn1 induced a predominant Th2 type whereas rLdPxn2 triggered a mixed Th1/Th2 with predominant Th1 type response. We also found that CpG ODN and GLA-SE enhance the production of a polarized Th1 type regardless of the initial response. In a recent study, we showed that priming with LdPxn1 DNA in the presence of murine granulocyte macrophage colony-stimulating factor (mGMCSF) and boosting with recombinant LdPxn1 protein stimulates multifunctional CD4^+^ T cells and protects mice against* L. major* infection [40]. It will be interesting to examine the protective effect of recombinant LdPxn1 and LdPxn2 proteins individually or combined with or without adjuvants against* Leishmania* infection.

## Figures and Tables

**Table 1:** The ratio of IFN-*γ*/IL-10 in lymph node cells of immunized mice.

	Antigen (10 *μ*g/mL)			Medium			ConA		
	Antigen	+CpG ODN	+GLA-SE	Antigen	+CpG ODN	+GLA-SE	Antigen	+CpG ODN	+GLA-SE
rLdPxn1	0	24.74	10.42	0	0	0	75.35	36.59	24.6
rLdPxn2	0	10.83	11.11	0	0	0	19.42	5.19	7.38

**Table 2:** The ratio of IFN-*γ*/IL-10 in spleen cells of immunized mice.

	Antigen (2 μg/mL)			Antigen (10 μg/mL)			Medium			ConA		
	Antigen	+CpG ODN	+GLA-SE	Antigen	+CpG ODN	+GLA-SE	Antigen	+CpG ODN	+GLA-SE	Antigen	+CpG ODN	+GLA-SE
rLdPxn1	0	2.88	0.53	0	3.78	2.03	0	0	0	22.04	34.62	28.91
rLdPxn2	3.37	18.3	7.75	12.82	10.88	7.55	0	0	0	60.24	80.84	34.76
